# Supplementary material for: The recombination landscape of introgression in yeast
Source: PLoS Genet. 2025 Feb 12;21(2):e1011585. doi: 10.1371/journal.pgen.1011585 (PMC11845044; doi:10.1371/journal.pgen.1011585)
Supplement: S1 Table — (DOCX) [file pgen.1011585.s012.docx]

| Chromosome | Natural count | Natural SE | Fermentation count | Fermentation SE |
| --- | --- | --- | --- | --- |
| 1 | 1.3958 | 0.1254 | 1.9574 | 0.0960 |
| 2 | 9.2083 | 0.3150 | 6.8298 | 0.2136 |
| 3 | 3.5000 | 0.1786 | 2.9362 | 0.1503 |
| 4 | 7.2292 | 0.2650 | 4.5319 | 0.1796 |
| 5 | 3.8750 | 0.1921 | 3.5106 | 0.1517 |
| 6 | 4.0208 | 0.1412 | 2.8936 | 0.1753 |
| 7 | 6.0208 | 0.2218 | 5.7234 | 0.2389 |
| 8 | 5.3750 | 0.2834 | 4.7021 | 0.1548 |
| 9 | 2.8333 | 0.2005 | 2.0638 | 0.1533 |
| 10 | 8.1667 | 0.2798 | 5.0638 | 0.2460 |
| 11 | 4.5208 | 0.2041 | 3.5319 | 0.1211 |
| 12 | 2.8958 | 0.1272 | 2.2340 | 0.1557 |
| 13 | 6.0625 | 0.2371 | 4.5957 | 0.1891 |
| 14 | 5.4167 | 0.2576 | 2.8936 | 0.1881 |
| 15 | 5.4583 | 0.2018 | 4.3404 | 0.2069 |
| 16 | 6.5625 | 0.2695 | 5.8511 | 0.2040 |
