# Supplementary material for: The recombination landscape of introgression in yeast
Source: PLoS Genet. 2025 Feb 12;21(2):e1011585. doi: 10.1371/journal.pgen.1011585 (PMC11845044; doi:10.1371/journal.pgen.1011585)
Supplement: S2 Table — (DOCX) [file pgen.1011585.s013.docx]

| Chromosome | Natural count | Natural SE | Fermentation count | Fermentation SE |
| --- | --- | --- | --- | --- |
| 1 | 0.3958 | 0.1020 | 0.7447 | 0.1376 |
| 2 | 2.3958 | 0.2340 | 1.7872 | 0.2948 |
| 3 | 1.5208 | 0.1657 | 0.6809 | 0.1294 |
| 4 | 2.2292 | 0.2442 | 3.1277 | 1.2153 |
| 5 | 1.0625 | 0.1746 | 0.4894 | 0.1249 |
| 6 | 1.8750 | 0.1944 | 1.4468 | 0.1943 |
| 7 | 2.2083 | 0.2325 | 2.1915 | 0.3658 |
| 8 | 0.5000 | 0.1074 | 1.4043 | 0.1891 |
| 9 | 1.3333 | 0.1745 | 2.1702 | 0.8392 |
| 10 | 2.6250 | 0.2787 | 3.2766 | 1.0574 |
| 11 | 1.3333 | 0.1719 | 1.1064 | 0.1616 |
| 12 | 0.2083 | 0.0592 | 0.5745 | 0.1451 |
| 13 | 2.4375 | 0.2695 | 3.3617 | 1.2270 |
| 14 | 1.8750 | 0.1921 | 7.9149 | 4.0521 |
| 15 | 1.5417 | 0.1929 | 3.2340 | 1.0061 |
| 16 | 1.9375 | 0.2256 | 1.3404 | 0.2001 |
