# Supplementary material for: The recombination landscape of introgression in yeast
Source: PLoS Genet. 2025 Feb 12;21(2):e1011585. doi: 10.1371/journal.pgen.1011585 (PMC11845044; doi:10.1371/journal.pgen.1011585)
Supplement: S3 Table — Each regression has either CO count, NCO count or NCO tract length as the response variable, and marker density as the independent variable. (DOCX) [file pgen.1011585.s014.docx]

| Chromosome | Response Variable | Intercept | Intercept p-value | Marker dens | Marker dens p-value | Adjusted R-squared |
| --- | --- | --- | --- | --- | --- | --- |
| 4 | CO | 0.4286 | 0 | 0.0000 | 0.3334 | 0.5002 |
| 4 | NCO | -0.1122 | 0 | 0.1623 | 0 | 0.9003 |
| 4 | Tract lenght | 4636.396 | 0 | 309.0741 | 0 | 0.6948 |
| 6 | CO | 5.2364 | 0 | 0.0016 | 0 | 0.2146 |
| 6 | NCO | 1.8130 | 0 | 0.0450 | 0.1426 | 0.0120 |
| 6 | Tract length | 3935.625 | 0 | -126.2340 | 0 | 0.2002 |
| 7 | CO | 0.1782 | 0.5991 | 0.6840 | 0 | 0.4360 |
| 7 | NCO | 0.1516 | 0 | 0.1508 | 0 | 0.8902 |
| 7 | Tract length | 265.9691 | 0 | -16.2048 | 0 | 0.3279 |
| 9 | CO | 2.0593 | 0 | 0.0066 | 0 | 0.2229 |
| 9 | NCO | 0.1689 | 0 | 0.1842 | 0 | 0.9053 |
| 9 | Tract length | 4187.5 | 0 | -257.4853 | 0 | 0.5112 |
| 10a | CO | 4.25 | 0 | 0 | 0.3334 | 0.4991 |
| 10a | NCO | 0.8418 | 0 | 0.0509 | 0 | 0.6643 |
| 10a | Tract length | 4432.434 | 0 | -193.1295 | 0 | 0.6062 |
| 10b | CO | 0.5730 | 0 | -0.0002 | 0.3334 | 0.0053 |
| 10b | NCO | -0.0357 | 0.0161 | 0.0812 | 0 | 0.8526 |
| 10b | Tract length | 5268.21 | 0 | -184.3647 | 0 | 0.7689 |
| 13 | CO | 1 | 0 | 0 | 0.3334 | 0.4993 |
| 13 | NCO | 0.1219 | 0.01 | 0.2993 | 0 | 0.8854 |
| 13 | Tract length | 6682.40 | 0 | -410.3597 | 0 | 0.7844 |
| 14 | CO | 1.8966 | 0 | 0.0583 | 0 | 0.2614 |
| 14 | NCO | -0.3230 | 0 | 0.2683 | 0 | 0.9562 |
| 14 | Tract length | 4239.71 | 0 | -276.0607 | 0 | 0.5724 |
| 15 | CO | 0.750 | 0 | 0 | 0.3334 | 0.5009 |
| 15 | NCO | 0.173 | 0.0004 | 0.3260 | 0 | 0.9130 |
| 15 | Tract length | 1796.619 | 0 | -128.5568 | 0 | 0.1834 |
