# Supplementary material for: The recombination landscape of introgression in yeast
Source: PLoS Genet. 2025 Feb 12;21(2):e1011585. doi: 10.1371/journal.pgen.1011585 (PMC11845044; doi:10.1371/journal.pgen.1011585)
Supplement: S4 Table — (DOCX) [file pgen.1011585.s015.docx]

| Chrom | CO correlation | CO correlation p-value | SNP correlation | SNP correlation p-value |
| --- | --- | --- | --- | --- |
| 1 | 0.6667 | 0.0353 | -0.0061 | 0.9867 |
| 2 | 0.2302 | 0.0650 | 0.0086 | 0.9459 |
| 3 | 0.3816 | 0.1604 | -0.3339 | 0.2238 |
| 4 | 0.2205 | 0.1553 | 0.3420 | 0.0248 |
| 5 | 0.5205 | 0.0054 | -0.0746 | 0.7117 |
| 6 | 0.5908 | 0.0030 | 0.2370 | 0.2762 |
| 7 | 0.2048 | 0.1580 | 0.0106 | 0.9425 |
| 8 | 0.2187 | 0.1695 | -0.2473 | 0.1190 |
| 9 | 0.1278 | 0.6774 | -0.5992 | 0.0305 |
| 10 | 0.2313 | 0.1510 | 0.2353 | 0.1438 |
| 11 | 0.5733 | 0.0006 | 0.2297 | 0.2060 |
| 12 | 0.4717 | 0.0231 | -0.2188 | 0.3157 |
| 13 | 0.2825 | 0.0735 | 0.1341 | 0.4032 |
| 14 | 0.8932 | 0.0028 | -0.6831 | 0.0618 |
| 15 | 0.4660 | 0.0048 | 0.3243 | 0.0573 |
| 16 | 0.4318 | 0.0027 | -0.0898 | 0.5528 |
