# Supplementary material for: The recombination landscape of introgression in yeast
Source: PLoS Genet. 2025 Feb 12;21(2):e1011585. doi: 10.1371/journal.pgen.1011585 (PMC11845044; doi:10.1371/journal.pgen.1011585)
Supplement: S5 Table — (DOCX) [file pgen.1011585.s016.docx]

| Chrom. | CO correlation | CO correlation p-value | SNP correlation | SNP correlation p-value |
| --- | --- | --- | --- | --- |
| 4 | 0.2118 | 0.6484 | 0.7143 | 0.0881 |
| 6 | 0.4000 | 0.7500 | -0.4000 | 0.7500 |
| 7 | 0.0000 | 1.0000 | -0.8660 | 0.3333 |
| 9 | -0.2547 | 0.5427 | 0.1928 | 0.6474 |
| 10 | -0.2071 | 0.5411 | 0.2727 | 0.4182 |
| 13 | 0.0000 | 1.0000 | 0.1539 | 0.8048 |
| 14 | 0.2274 | 0.2269 | -0.3782 | 0.0393 |
| 15 | 0.2000 | 0.9167 | 0.8000 | 0.3333 |
