# Supplementary material for: The recombination landscape of introgression in yeast
Source: PLoS Genet. 2025 Feb 12;21(2):e1011585. doi: 10.1371/journal.pgen.1011585 (PMC11845044; doi:10.1371/journal.pgen.1011585)
Supplement: S6 Table — (DOCX) [file pgen.1011585.s017.docx]

| Chromosome | Natural  mean | Natural  SE | Fermentation  mean | Fermentation  SE | t-test p-value |
| --- | --- | --- | --- | --- | --- |
| 1 | 0.2615 | 0.0140 | 0.2651 | 0.0132 | 0.8498 |
| 2 | 0.4264 | 0.0068 | 0.3993 | 0.0090 | 0.0172 |
| 3 | 0.3101 | 0.0121 | 0.3104 | 0.0131 | 0.9848 |
| 4 | 0.4166 | 0.0067 | 0.3728 | 0.0100 | 0.0003 |
| 5 | 0.3606 | 0.0106 | 0.3267 | 0.0125 | 0.0391 |
| 6 | 0.3672 | 0.0106 | 0.3265 | 0.0130 | 0.0158 |
| 7 | 0.4034 | 0.0085 | 0.3956 | 0.0091 | 0.5307 |
| 8 | 0.3680 | 0.0108 | 0.3803 | 0.0096 | 0.3968 |
| 9 | 0.3124 | 0.0133 | 0.2269 | 0.0135 | 8.75x10^-6^ |
| 10 | 0.4061 | 0.0074 | 0.3655 | 0.0105 | 0.0018 |
| 11 | 0.3674 | 0.0103 | 0.3388 | 0.0114 | 0.0634 |
| 12 | 0.3583 | 0.0115 | 0.2699 | 0.0141 | 1.9x10^-6^ |
| 13 | 0.3784 | 0.0098 | 0.3896 | 0.0099 | 0.4228 |
| 14 | 0.3758 | 0.0106 | 0.2163 | 0.0114 | <2.2x10^-16^ |
| 15 | 0.4064 | 0.0076 | 0.3582 | 0.0112 | 0.0004 |
| 16 | 0.3991 | 0.0089 | 0.3808 | 0.0103 | 0.1794 |
