# Supplementary material for: The recombination landscape of introgression in yeast
Source: PLoS Genet. 2025 Feb 12;21(2):e1011585. doi: 10.1371/journal.pgen.1011585 (PMC11845044; doi:10.1371/journal.pgen.1011585)
Supplement: S7 Table — (DOCX) [file pgen.1011585.s018.docx]

| Chromosome | Natural  mean | Natural  SE | Fermentation  mean | Fermentation  SE | t-test p-value |
| --- | --- | --- | --- | --- | --- |
| 4 | 0.1539 | 0.0131 | 0.0161 | 0.0059 | <2.2x10^-16^ |
| 6 | 0.0577 | 0.0111 | 0.0175 | 0.0058 | 0.0015 |
| 7 | 0.2678 | 0.0075 | 0.1551 | 0.0155 | 2.78x10^-10^ |
| 9 | 0.1826 | 0.0146 | 0.0393 | 0.0087 | 1.15x10^-15^ |
| 10a | 0.0604 | 0.0106 | 0.0123 | 0.0043 | 3.46x10^-5^ |
| 10b | 0.2017 | 0.0152 | 0.0075 | 0.0029 | <2.2x10^-16^ |
| 13 | 0.1040 | 0.0140 | 0.0193 | 0.0055 | 4.65x10^-8^ |
| 14 | 0.3364 | 0.0126 | 0.1513 | 0.0120 | <2.2x10^-16^ |
| 15 | 0.1596 | 0.0153 | 0.0242 | 0.0056 | 6.81x10^-15^ |
