# Supplementary material for: The recombination landscape of introgression in yeast
Source: PLoS Genet. 2025 Feb 12;21(2):e1011585. doi: 10.1371/journal.pgen.1011585 (PMC11845044; doi:10.1371/journal.pgen.1011585)
Supplement: S8 Table — (DOCX) [file pgen.1011585.s019.docx]

| Strain number | Strain name | Genotype | Isolation source | Isolation location | Cross | NCBI SRA | Citation | Obtained from |
| --- | --- | --- | --- | --- | --- | --- | --- | --- |
| yCSH347 | yHCT78 |  | Bark of Quercus acutissima | Chaumette Vineyard, Ste. Genevieve, Missouri |  | SRR1119189 | Almeida et al 2014 [72] | Chris Hittinger |
| yCSH345 | UCD61-137 |  | Drosophila pseudoobscura | Berryessa Hills, California |  | SRR1119180 | Almeida et al 2014 [72] | Portugese Yeast Culture Collection (PYCC 6878) |
| yCSH561 | DBVPG 7787 |  | Wine | Slovakia |  | SRR1119199 | Almeida et al 2014 [72] | Portugese Yeast Culture Collection (PYCC6876) |
| yCSH35 | GM14 |  | Fermenting grape must | France |  | SRR1119200 | Almeida et al 2014 [72] | Portugese Yeast Culture Collection (PYCC 6892) |
| yCSH833 | GM14 | HOdelta0::kanMX MATa |  |  | European fermentation |  | This study |  |
| yCSH836 | DBVPG 7787 | HOdelta0::kanMX MATx |  |  | European fermentation |  | This study |  |
| yCSH837 | UCD61-137 | HOdelta0::kanMX MATa |  |  | North American natural |  | This study |  |
| yCSH840 | yHCT78 | HOdelta0::kanMX MATx |  |  | North American natural |  | This study |  |
| Plasmid strain number |  |  |  |  |  |  |  |  |
| pCSH2 |  | pFA6a-TEF2Pr-dTomato-ADH1-Primer-KANMX6 |  |  |  |  |  |  |
