# Supplementary material for: The recombination landscape of introgression in yeast
Source: PLoS Genet. 2025 Feb 12;21(2):e1011585. doi: 10.1371/journal.pgen.1011585 (PMC11845044; doi:10.1371/journal.pgen.1011585)
Supplement: S9 Table — (DOCX) [file pgen.1011585.s020.docx]

| Chromosome | Start | End | Size | Type |
| --- | --- | --- | --- | --- |
| 5 | 294582 | 306485 | 11903 | amplification |
| 7 | 840000 | 859500 | 19500 | amplification |
| 15 | 414372 | 416662 | 2290 | amplification |
