# Supplementary material for: The recombination landscape of introgression in yeast
Source: PLoS Genet. 2025 Feb 12;21(2):e1011585. doi: 10.1371/journal.pgen.1011585 (PMC11845044; doi:10.1371/journal.pgen.1011585)
Supplement: S10 Table — (DOCX) [file pgen.1011585.s021.docx]

| Chromosome | Start Position | End position |
| --- | --- | --- |
| 4 | 866500 | 983774 |
| 6 | 1 | 65500 |
| 7 | 1 | 53500 |
| 9 | 158500 | 298500 |
| 10 | 234500 | 288500 |
| 10 | 301500 | 428500 |
| 13 | 26500 | 103500 |
| 14 | 18500 | 586500 |
| 15 | 367500 | 434500 |
